# Structure-Guided Design of Therapeutic Antibodies Targeting SARS-CoV-2 Omicron Variants

**DOI:** 10.21203/rs.3.rs-9917568/v1

**Published:** 2026-06-24

**Authors:** Jesper Pallesen, Jianqiu Du, Yuanhan Wu, Sukanya Ghosh, Kelly Bayruns, Roopak Sadeesh, David Weiner

**Affiliations:** The Wistar Institute; The Wistar Institute; The Wistar Institute; The Wistar Institute; The Wistar Institute; The Wistar Institute; The Wistar Institute

## Abstract

The ongoing evolution of SARS-CoV-2, particularly the emergence of Omicron subvariants, compromised the effectiveness of many therapeutic antibodies. In this study, we employed a structure-guided computational design strategy to systematically optimize the COV2–2196 antibody for improved neutralization of Omicron variants. Through iterative rounds of computational design and experimental validation, we identified key paratope mutations that restored and enhanced antibody binding and neutralization potency against resistant viral strains. Cryo-EM structural analysis revealed the molecular basis for these improvements, highlighting how targeted modifications can accommodate epitope changes introduced by viral evolution. Our approach demonstrates that effective antibody optimization can be achieved using accessible computational resources, providing a practical framework for rapid therapeutic development. These findings underscore the potential of structure-based design to address challenges posed by viral antigenic drift and support the development of broadly effective antibody therapeutics for emerging infectious diseases.

## Introduction

By the end of 2019, a coronavirus disease (COVID-19), caused by Severe Acute Respiratory Syndrome Coronavirus-2 (SARS-CoV-2), had rapidly spread worldwide^1,2^. The COVID-19 pandemic highlighted the critical role of monoclonal antibody (mAb)-based therapeutics in both the prevention and treatment of infectious diseases^3^. Several mAb drug products demonstrated strong efficacy in preventing COVID-19 as well as lowering severity of illness in hospitalized COVID-19 patients and were granted emergency use authorization (EUA) by the U.S. FDA, contributing to significant reductions in viral load, hospitalization, and mortality^4–9^. Among these, Evusheld—a combination of tixagevimab and cilgavimab derived from COV2–2196 (2196WT) and COV2–2130 (2130WT), respectively—was the first and only antibody therapy approved for pre-exposure prophylaxis in immunocompromised individuals for whom vaccination may be insufficient^4^. However, the emergence of the SARS-CoV-2 Omicron variant BA.1.159 (BA.1), first reported in November 2021, challenged the effectiveness of Evusheld and similar therapies^9,10^. BA.1 was rapidly replaced by subvariant BA.2 in late December 2021. BA.2 quickly became the dominant global variant and substantially reduced or eliminated the neutralizing activity of many authorized mAbs. Evusheld exhibited a 10 to 100-fold reduction in neutralization potency^11–13^. Particularly, the COV2–2196-based component tixagevimab exhibited a 1000-fold reduction in BA.2 neutralization in comparison to the early USA/WA1/2020 (WA) strain^11,14^. Come April 2022, variants BA.4 and BA.5—carrying virtually identical Spike protein Receptor Binding Domains (RBDs)—had emerged and replaced BA.2 as the dominant variants^15^. Additional BA.4 and BA.5 point mutations included Spike protein F486V and a point mutation reversal R493Q which resulted in further reduced COV2–2196 neutralization potency^16^.

Development of computational protein design has proven to facilitate optimization of antibodies targeting SARS-CoV-2^17,18^. Thus, we hypothesized that by employing computational design strategies, we would be able to restore 2196WT antibody neutralization to Omicron escape variants. Our pipeline implemented structure-guided iterative computational design combined with experimental validation and we used Rosetta InterfaceAnalyzer to obtain ΔΔG calculations for down-selecting our computational designs. We identified multiple re-designed COV2–2196 antibody versions establishing neutralization toward Omicron SARS-CoV-2 variants similar to the original COV2–2196 antibody against the SARS-CoV-2 WA strain. We provide cryo-EM structures of those top-candidate neutralizing antibody designs in complex with soluble trimeric Spike proteins to explain the molecular basis of the rescued neutralization potency and to provide a structural basis for further antibody design. To dramatically reduce computational resource requirement, our method implements cryo-EM structure determination of designed top-candidate antibodies and thereby allows universal and precise iterative antibody design to recover neutralization potency from “non-neutralizer” to “neutralizer” as exemplified by our re-design of COV2–2196. This strategy enables computationally efficient antibody design aiming to overcome challenges of future or currently circulating viruses.

## Results

### Cryo-EM analysis of BA.1 Spike protein interactions with 2196 and 2130

We attempted to solve a cryo-EM structure of soluble trimeric BA.1 Spike protein (BA.1-S) with Fab from antibody COV-2196 (2196WT) and wildtype Fab from antibody COV-2130 (2130WT). We sought to complex soluble trimeric BA.1-S with Fabs from 2196WT and 2130WT by mixing the components at molar a ratio of 1:9:9, respectively, followed by size-exclusion chromatography (Extended Data Fig. 1a). As expected—in line with the drastically reduced 2196 affinity to BA.1-S^13^—the BA.1-S complex exhibited only a moderate shift in the SEC chromatogram. The sample was imaged by negative-stain electron microscopy and the resulting 2D class averages showed 2130 Fab binding but no binding of 2196 Fab (Extended Data Fig. 1a, b).

We hypothesized that 2196 IgG would have a slightly higher affinity to BA.1-S due to the intrinsic avidity effect involved in IgG binding compared to Fab binding. We thus complexed BA.1-S with 2130 IgG, 2196 IgG or with both 2130 and 2196 IgGs followed by SEC purification. The resulting SEC chromatograms demonstrated that 2130 IgG as well as 2196 IgG were capable of binding to BA.1-S with high enough affinity for SEC purification and cryo-EM structural analyses (Extended Data Fig. 1a). However, complexation of both 2130 and 2196 IgGs to BA.1-S resulted in aggregation (agglutination) and this sample was deemed unsuitable for characterization by cryo-EM (Extended Data Fig. 1a).

The purified BA.1-s/2130IgG and BA.1-S/2196IgG samples underwent cryo-EM structure determination, and we obtained cryo-EM density maps with a global resolution of 2.9 Å (BA.1-S/2130IgG) and 3.6 Å (BA.1-S/2196IgG) (Extended Data Fig. 1c, Extended Data Table 1). The RBD regions that were presumably bound to 2130IgG or 2196IgG were not well-resolved in either cryo-EM density map. We therefore employed a local density map refinement strategy and we obtained a well-resolved local density map corresponding to BA.1-S RBD and the variable domains V_H_ and V_L_ of 2130IgG at a resolution of 3.2 Å (Extended Data Fig. 1c, Extended Data Table 1). In addition, this local BA.1-RBD/2130WT density map exhibited a well-resolved, unoccupied 2196 epitope. Unfortunately, we were unable to obtain a local density map of BA.1-S RBD/2196IgG.

### Structure-guided computational design strategy

**Figure 1:**
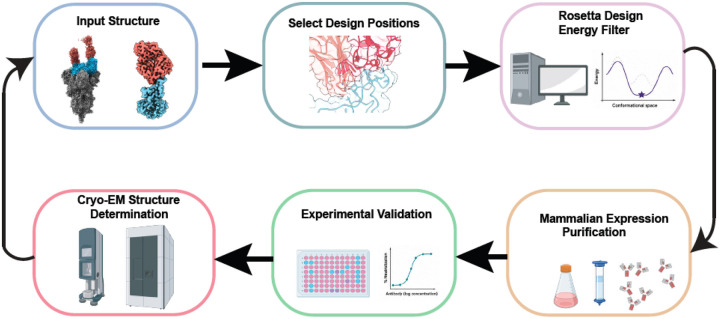
Structure-guided design workflow. In general, an input structure is applied as basis followed by design position selection. Rosetta multi-position design is then performed followed by Rosetta energy filtering. Top antibody design candidates are then expressed and purified. Experimental characterization is performed against SARS-CoV-2 Omicron variants. Validated antibody designs are then used to determine cryo-EM structures that are used as basis for next design iteration.

We applied our computational design pipeline to perform three Iterations of design (Fig. 1 and Table 1). In our Iteration 1, we aimed to provide a cryo-EM structure of BA.1-S in complex with a redesigned 2196 antibody that could serve as basis for further 2196 design optimization. These Iteration 1 designs resulted in a stable complex of BA.1-S/2196-G50L-S93F that we used for precise cryo-EM structure determination. Using this structure to analyze the BA.1-S/2196-G50L-S93F epitope/paratope interface, we identified 11 amino acid positions for design in Iteration 2. Iteration 2 aimed to provide 2196-based antibody designs that recovered lost BA.1 and BA.2 neutralization properties. We modeled our Iteration 2 lead candidate 2196 design, Ab#10, with BA.4-S and used this model as basis for our Iteration 3 designs. Here, we aimed to design a 2196 variant that recovered lost neutralization against BA.4 and BA.5 virus variants. Iteration 3 employed single mutation scanning and we identified an antibody variant that fully rescued 2196 neutralization potency against the SARS-CoV-2 Omicron BA.4 variant.

### Iteration 1 design

Both BA.1 and BA.2 RBD proteins harbor 12 shared mutations in the receptor-binding domain (RBD), including G339D, S373P, S375F, K417N, N440K, S477N, T478K, E484A, Q493R, Q498R, N501Y and Y505H, compared to the ancestral strain^15^. Among these shared mutations, S477N, T478K, E484A and Q493R contributed to impaired neutralization potency for several therapeutic antibodies^19^. 2196WT partially lost its neutralization potency against both BA.1 and BA.2 strains, while 2130WT exhibited reduced neutralization potency against BA.1 but partially recovered neutralization against BA.2^13^.

**Figure 2:**
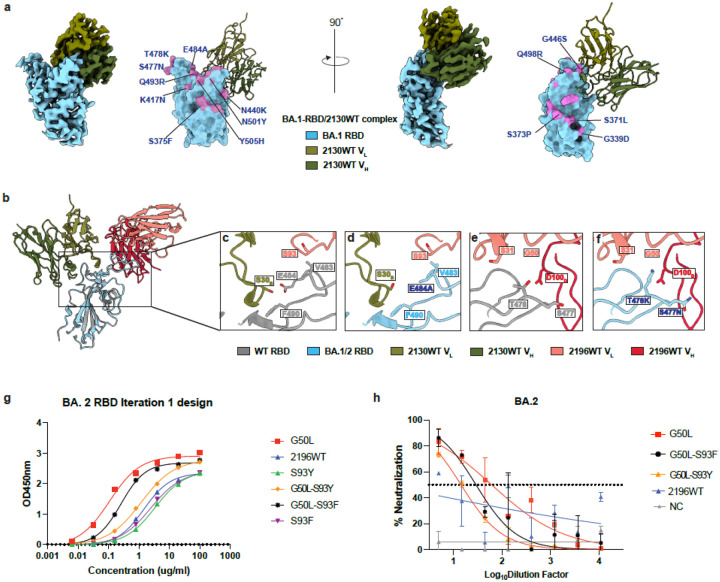
Iteration 1 design improved 2196 neutralization against BA.2 pseudovirus. a. Structural basis of Iteration 1 design. Views of the BA.1-RBD/2130WT local cryo-EM density map and model. BA.1-RBD, 2130WT VL and VH are colored as visualized in panel. BA.1-RBD is shown as surface and mutations from WA-RBD is colored in pink. 2130WT VL and VH are shown as ribbon. b-f. Amino acid positions chosen for Iteration 1 design. b. 2196WT was modeled onto our BA.1-RBD/2130WT structure. c and e. Positional contacts between WA-RBD to 2196WT and 2130WT. d and f. Key mutations on BA.1-RBD affects the 2130WT and 2196WT interactions. g and h. Iteration 1 design experimental validation. g. ELISA binding curves of Iteration 1 designed antibodies against BA.1/2-RBD. Lines represent a four-parameter logistic regression model fitted in GraphPad Prism with two technical replicates. h. Pseudovirus neutralization of Iteration 1 designed antibodies against BA.2 pseudovirus. Each curve and data point are colored as visualized in the panels. NC: Negative control antibody, 8ANC195 (anti-HIV1). Curves represent a fourparameter logistic regression model fitted in GraphPad Prism with two technical replicates. Neutralization data has been repeated in 3 independent assays.

We combined our local BA.1-S/2130WT density map (Fig. 2a)—containing high-resolution information of the BA.1-S epitope recognized by 2196—with our structure of WA-S/2196WT^19^ which allowed us to model the interaction of BA.1-S with 2196WT (Fig. 2b–f). Detailed analysis of our model revealed that specific mutations on BA.1-S RBD had disrupted critical interactions with the antibodies. The E484A mutation shortened the side chain and changed its chemistry to hydrophobic, eliminating a hydrogen bond (h-bond) with S30_B_ on the CDRL1 region of 2130WT (Fig. 2c,d). Similarly, the BA.1 T478K mutation broke an h-bond with D100_D_ on the CDRH3 of 2196WT (Fig. 2e,f). Additional structural observations included G50 on the CDRL2 of 2196WT contributing a void volume near T478K (Fig. 2c,d), while the side chain of S93 on 2196WT CDRL3 engaged V483 through Cβ interactions (Fig. 2e,f). We hypothesized that mutations at G50 and S93 of 2196WT and S30_B_ of 2130WT could enhance interactions with the BA.1 RBD by introducing favorable hydrophobic interactions or filling the void volume observed in our model. Using Rosetta, we conducted multi-position Rosetta design and obtained 120 designed variants of 2130 (focusing on S30_B_) and 120 designed variants of 2196 (targeting S93 and G50). Rosetta ΔΔG energy based ranking identified G50L and S93Y or S93F for 2196WT as favorable substitutions, as well S30_B_G for 2130WT (Extended Data Fig. 2a). Subsequent ELISA binding assays showed that 2196-G50L, as well as combinations with S93Y or S93F (G50L-S93Y and G50L-S93F), exhibited improved binding to both BA.1/2 and BA.4/5 RBDs (Fig. 2g and Extended Data Fig. 2b). Comparing with 2196WT, 2196-G50L, 2196-G50L-S93Y and 2196-G50L-S93F recovered neutralization potency to BA.2 pseudovirus with half-maximal inhibitory concentration (IC50) of 7.31 ng/mL, 23.5 ng/mL and 10.94 ng/mL, respectively (Fig. 2h and Extended Data Fig. 2c).

**Figure 3:**
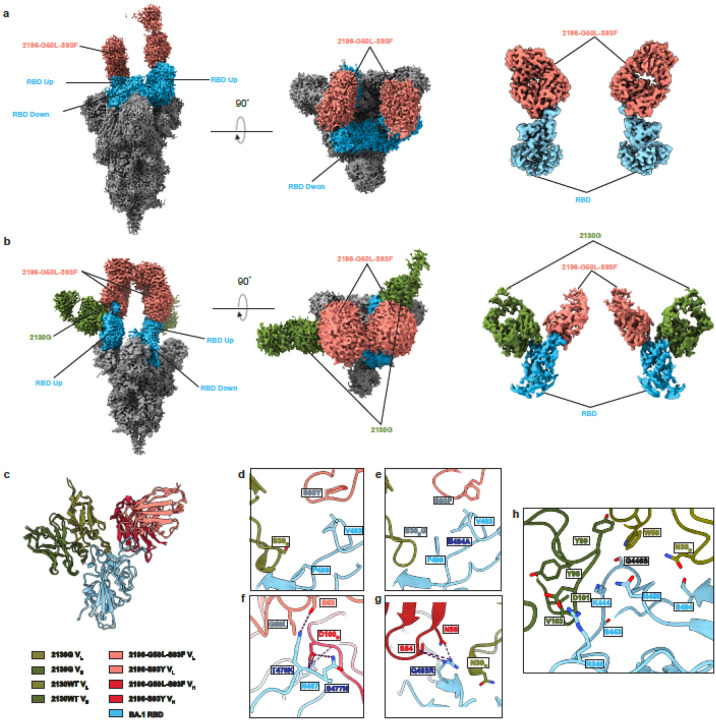
Cryo-EM structure of Iteration 1 designed antibodies in complex with BA.1-S. a. Cryo-EM density map of BA.1-S/2196-G50L-S93F complex. b. Cryo-EM density map of BA.1-S/2130-S30BG/2196-G50L-S93F complex. Corresponding locally refined density maps are shown next to the global density maps. Resolved densities for 2196-G50L-S93F IgG, 2130WT Fab, 2130-S30BG Fab and RBD are colored in salmon, green and blue. c-h. Epitope interface analysis. Hydrogen bounds were colored in purple and dashed lines. c. Superimposed BA.1-RBD/2130WT/2196-G50L-S93Y and BA.1-RBD/2130-S30BG/2196-G50L-S93F complexes. Each chain is colored as shown in figure panel. d and e. Interactions formed by S93Y or S93F and S30BG mutations from Iteration 1 design. BA.1 RBD mutations are labeled in deep blue. f. Detailed contacts formed surrounding G50L from Iteration 1 design and interactions surrounding BA.1-RBD T478K and S477N mutations. g. Interactions surrounding BA.1-S RBD mutation Q493R. h. Interactions maintained by between BA.1-RBD and 2130WT.

We attempted to obtain structures of our designs to use as basis for our Iteration 2 designs. Thus, we solved cryo-EM structures of 2196-S93Y or G50L-S93F IgG with or without 2130WT or 2130-S30_B_G in complex with BA.1-S, resulting in BA.1-S/2196-S93Y, BA.1-S/2196-G50L-S93F, BA.1-S/2196-S93Y/2130WT and BA.1-S/2196-G50L-S93F/2130-S30_B_G cryo-EM density maps (Fig. 3, Extended Data Fig. 3, Extended Data Fig. 6 and Extended Data Table 1). The global density map resolution of BA.1-S/2196-S93Y was 2.8Å, BA.1-S/2196-G50L-S93F 3.6Å, BA.1-S/2196-S93Y/2130WT 3.1Å and BA.1-S/2196-G50L-S93F/2130-S30_B_G 3.6Å (Fig. 3, Extended Data Fig. 3, Extended Data Fig. 6 and Extended Data Table 1). In all global density maps obtained, RBD regions and their antibody binding partners were not well-resolved. We therefore employed a local density map refinement strategy and we obtained well-resolved RBD density maps corresponding to BA.1-S RBD and the variable domains V_H_ and V_L_ of our designed antibodies. Local density map resolutions were as follows: BA.1-S/2196-S93Y 3.1 Å, BA.1-S/2196-G50L-S93F 3.6 Å, BA.1-S/2196-S93Y/2130WT 3.9 Å and BA.1-S/2196-G50L-S93F/2130-S30_B_G 4.3 Å (Fig. 3, Extended Data Fig. 3, Extended Data Fig. 6 and Extended Data Table 1). In all four structures, two BA.1-S RBDs were in “up” positions and one RBD was in a “down” position. RBD positions were thus unchanged regardless of complexation of 2130WT or 2130-S30_B_G Fabs (Fig. 3a, b)^4^.

Our subsequent detailed analysis of antibodies-BA.1-S paratope/epitope interfaces revealed that multiple new stabilizing interactions had formed (Fig. 3c–h). We observed the formation of hydrophobic interactions between the side chain of 2196 CDRL3 S93Y or S93F of 2196-S93Y or 2196-G50L-S93F, respectively, with BA.1-S RBD V483. We further observed hydrophobic interactions between CDRL2 G50L of 2196-G50L-S93F with the base of the RBD T478K side chain (Fig. 3d–f). Moreover, the interaction of G50L with the side chain of T478K locks the rotamer of T478K to form an h-bond with S53 of 2196-G50L-S93F (Fig. 3f). We also observed that the BA.1-S RBD F490 side chain flipped up to form an additional hydrophobic interaction with S30_B_G of 2130-S30_B_G CDRL1 (Fig. 3d, e).

**Figure 4:**
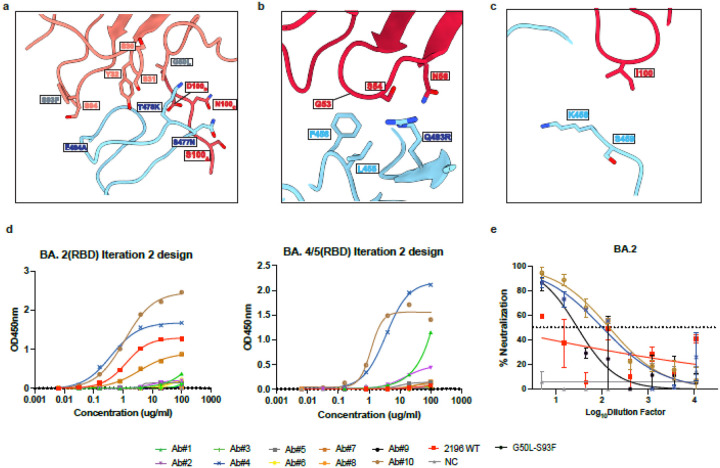
Iteration 2 designed antibodies further improve neutralization against the BA.2 variant. a-c. 2196 VL and VH amino acid positions for Iteration 2 design identified from our Iteration 1 BA.1-S/2196- G50L-S93F structure. Positions mutated in Iteration 1 design are labeled in grey on 2196-G50L-S93F. BA.1-RBD mutations are labeled in deep blue. d. ELISA assays of Iteration 2 top antibody designs tested against BA.1/2 and BA.4/5 RBD. Curves are fitted by four-parameter logistic regression models in GraphPad Prism with two replicates. e. BA.2 pseudovirus neutralization by antibodies Ab#4 and Ab#10. Curves represent a four-parameter logistic regression model fitted in GraphPad Prism with two replicates. Neutralization data was obtained from 3 independently repeated assays.

In addition to the increased binding stability introduced by our Iteration 1 designs of 2196 and 2130, our structure ensemble also explains the partially retained neutralization ability of the 2196WT/2130WT antibody cocktail^15,19^. Compared to interactions with WA-S, h-bond interactions were maintained between 2196 CDRH3 D100_D_ with the peptide bond of BA.1-S T478K, S477N and N487 side chains (Fig. 3f). WA-S Q493 interacts with 2196 CDRH2 S54 by h-bonding; BA.1-S Q493R also engages 2196 S54 and N56 by h-bond (Fig. 3g). While most of the h-bonds between 2130 and BA.1-S were maintained, WA-S RBD G446 hydrophobic interaction with 2130 W50 was replaced by an h-bond of BA.1-S G446S with W50 (Fig. 4h).

In summary, our Iteration 1 design of 2196WT resulted in 2196-G50L, 2196-G50L-S93Y and 2196-G50L-S93F exhibiting improved binding and neutralization potency against BA.2 strains. Our cryo-EM structure ensemble demonstrated key interactions that contribute to the improved binding and neutralization potency of our designed 2196-G50L, 2196-G50L-S93Y and 2196-G50L-S93F antibody variants.

### Iteration 2 design

To improve our design of antibody 2196, we used our Iteration 1 structure of BA.1-S/2196-G50L-S93F as a basis for our Iteration 2 antibody engineering. We performed restricted multi-position Rosetta design. Specifically, we focused on the mutations on BA.1-S RBD that impact the 2196 epitope/paratope interface, but we also attempted to introduce additional interactions. We first identified 2196 amino acids within 6Å of the mutated positions of BA.1-RBD. For RBD T478K, we selected amino acids S31, Y32 and G50L of the 2196-G50L-S93F light chain. Similarly, for RBD E484A, which has hydrophobic properties, we restricted 2196-G50L-S93F light chain amino acid positions S93F and S94 to hydrophobic computational design only. Since S477N still maintains its hydrophilicity, we restricted 2196 heavy chain positions S100_A_ and D100_D_ to hydrophilic computational design. The 2196-G50L-S93F N100_C_ side chain is positioned out and away from the RBD so we excluded N100_C_ as a design position (Fig. 3a). Additionally, for RBD Q493R, with hydrophobic residues F456 and I455 surrounding it, we chose 2196 heavy chain amino acid positions N56, S54 and G53 for design, where G53 was restricted to hydrophobic design and S54 and N56 were allowed all-amino-acid design excluding cysteine and proline (Fig. 3b). For adding extra interactions, we noticed that the CDRH3 tip of 2196-G50L-S93F was not involved in interactions with BA.1-S. We thus included amino acid position I100 for hydrophilic amino acid design as surrounding BA.1-S K458 and S459 have hydrophilic properties (Fig. 3c).

We conducted multi-position Rosetta design and obtained 3000 engineered 2196 antibody candidates. We ranked these designs based on calculated ΔΔG and selected our top 10 designs for further characterization. We expressed these 10 2196 antibody designs as IgGs in Expi293 cells (Fig. 3a and Extended Data Fig. 4a). ELISA binding assays showed that Ab#4 and Ab#10 (10 amino acid substitutions in each; Extended Data Fig. 4c) exhibit improved binding to BA.1/2 RBD. We further note that Ab#8 was the only design breaking the h-bond to RBD BA.1-S Q493R by introducing an S54L substitution (Extended Data Fig. 4c). This resulted in complete loss of affinity to BA.2-RBD (Fig. 4). Pseudovirus neutralization assays demonstrated improved neutralization potency of Ab#4 and Ab#10 compared to 2196WT as well as their parental antibody 2196-G50L-S93F from Iteration 1. The IC50 of Ab#4 and Ab#10 against BA.2 pseudovirus were 3.51 ng/mL and 2.56 ng/mL, respectively (Fig. 4d, e and Extended Data Fig. 4d). Surprisingly, both Ab#4 and Ab#10 exhibited partial binding ability to BA.4 RBD (or equivalently BA.5 RBD as they virtually share RBD composition) (Fig. 4d). Since the designs on 2196 light chain amino acid positions S93 and G50 differed between Iteration 1 and Iteration 2 (S93Y or S93F and G50L in Iteration 1 but S93G and G50Y in Iteration 2), we investigated whether a combination of the Iteration 1 and 2 designs would further improve Ab#4 or Ab#10 binding to BA.1/2 and BA.4/5 RBDs. We therefore introduced G93F and Y50L mutations on Ab#4 and on Ab#10. ELISA binding showed that G93F and Y50L reduced binding of both Ab#4 and Ab#10 against both BA.1/2 and BA.4/5 RBDs (Extended Data Fig. 4e).

In summary, by employing multi-position Rosetta design across the BA.1/2-RBD/2196-G50L-S93F epitope/paratope interface, we were able to identify Ab#4 and Ab#10 that exhibit improved binding ability over 2196WT. Both Ab#4 and Ab#10 showed improved neutralization potency toward BA.1 pseudovirus as well and they acquired low-affinity binding ability toward BA.4/5 RBD.

### Iteration 3 design

**Figure 5:**
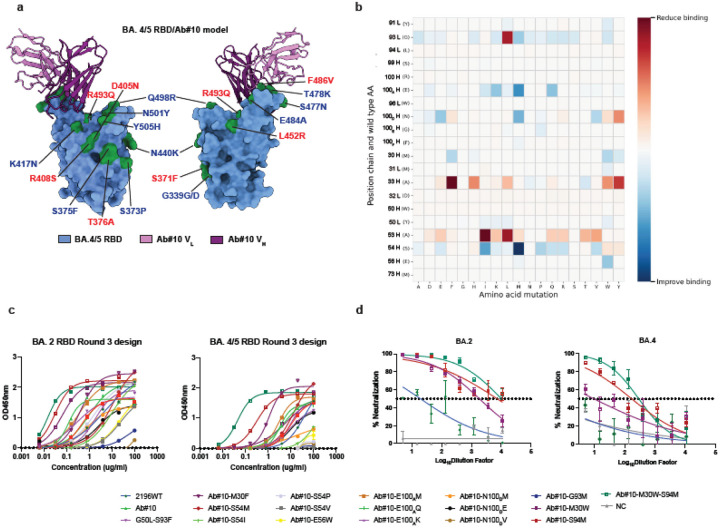
Iteration 3 designed antibody restores neutralization potency against BA.4 variant. a. BA.4/5-RBD/Ab#10 model. BA.4/5-RBD is rendered as surface. Each chain is colored as described in panel. Ab#10 VL and VH are shown as ribbon. Mutations that are identical to BA.1/2-RBD are labeled in deep blue and those that are unique to BA.4/5 are labeled in red. b. Single mutation scanning heatmap. Mutations that favorably reduce binding energy are colored as blue shades in accordance with legend. c. ELISA binding of Iteration 3 designed antibodies against BA.1/2 or BA.4/5 RBD. Curves represent a four-parameter logistic regression model fitted in GraphPad Prism with two replicates. d. Pseudoviral neutralization of top Iteration 3 designed antibodies against BA.2 and BA.5 pseudoviruses. Curves represent a four-parameter logistic regression model fitted in GraphPad Prism with two replicates. Neutralization data was repeated in 3 assays.

Although Ab#10 exhibited restored neutralization potency of the BA.2 pseudovirus variant, it failed to neutralize the BA.4 pseudovirus variant (Extended Data Fig. 4b). To redesign Ab#10 to target BA.4/5 variants, we performed our Iteration 3 design based on Ab#10. Both BA.4 and BA.5 RBDs harbored unique mutations S371F, T376A, D405N, R408S, L452R and F486V as well as an R493Q mutation reversal compared to BA.1/2 RBD. As noted earlier, 2196WT as well as our successful Iteration 1 and Iteration 2 2196 designs preserved the h-bond to WA-RBD 493Q or BA.1/2-RBD Q493R. We thus reasoned that F486V is the key BA.4/5 mutation as it disrupts a hydrophobic core formed with 2196^11^. We therefore computationally modeled the BA.4/5 substitutions R493Q and F486V as well as our Ab#10 amino acid substitutions (our best design from Iteration 2) onto our BA.1-RBD/2196-G50L-S93Y structure from Iteration 1 and obtained a model of BA.4-RBD/Ab#10. We used this model as basis for our Iteration 3 design (Fig. 5a). We then scanned the residues on the entire epitope/paratope interface of this model and identified 20 amino acid design positions. To limit computational costs, we implemented single-position NNK scanning using PyRosetta to systematically mutate each residue position at the interface and identify amino acid substitutions with favorable per-residue ΔΔG values. We visualized the results as a heatmap, highlighting mutations that enhance or reduce binding affinity, providing a guide for engineering antibody-antigen interactions (Fig. 5b). Single mutations contributing ΔΔG values of more than 1 Rosetta Energy Unit (REU) were chosen to engineer Iteration 3 antibody design candidates. In addition to ΔΔG-identified amino acid substitutions, we inspected the S94L amino acid position as S94L established a hydrophobic core with E484A and Y489 of the BA.1/2/4/5 RBDs (Extended Data Fig. 5a). We substituted methionine as our design candidate (S94M) since we reasoned that bulky hydrophobic side chains such as phenylalanine or tryptophan were likely to clash with Y489 (Extended Data Fig. 4a). Consequently, we compiled 16 Iteration 3 2196 antibody design candidates that we expressed as IgGs in Expi293 cells and purified. We used ELISA assays to determine the binding abilities of our design candidates to BA.4/5 RBD or BA.1/2 RBD. ELISA data showed that 10 out of our 16 candidates showed improved BA.1/2 RBD binding abilities compared to 2196WT, 5 compared to 2196-G50L-S93F (from Iteration 1) and 3 candidates compared to Ab#10 (from Iteration 2). For our target BA.4/5 RBD, 10 of our candidates showed improved binding compared to 2196WT, 9 compared to 2196-G50L-S93F and 5 candidates showed improved binding ability to BA.4/5 RBD compared to Ab#10 (Fig. 5c). Overall, our two designs Ab#10-S94M and Ab#10-M30W-S94M showed the most improved binding across BA.1/2/4/5 RBDs (Fig. 5c).

We next tested the neutralization potency of our best Iteration 3 design candidates against both BA.2 and BA.4 pseudoviruses. Our results showed that the Ab#10-M30W-S94M design showed drastically improved neutralization potency against BA.4 and retained outstanding neutralization potency to BA.2 pseudovirus as well (Fig. 5d). The calculated IC50 of Ab#10-M30W-S94M to BA.2 and BA.4 pseudoviruses were 0.05 ng/mL and 1.29 ng/mL, which is comparable to the 2196WT to WA pseudovirus (0.7 ng/mL) (Extended Data Fig. 5c)^4,20^. Notably, Ab#10-M30W-S94M also maintained high potency against WA pseudovirus with IC50 value of 0.52 ng/mL (Extended Data Fig. 5b, c; similar to 2196WT).

**Figure 6:**
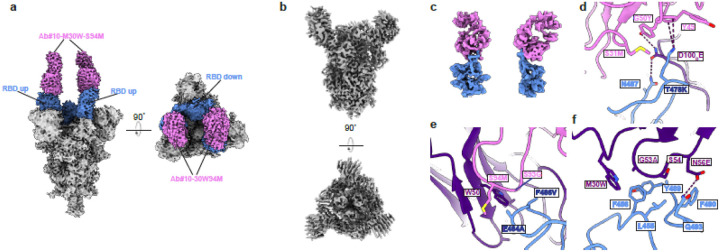
Cryo-EM structure of BA.4-S/Ab#10-M30W-L94M IgG complex. a. Global cryo-EM density map of BA.4-S/Ab#10-M30W-L94M IgG complex. Ab#10-M30W-L94M and BA.4-S RBD colors are shown in panel. b-f. Local density map and paratope-epitope interface analysis. Hydrogen bounds are shown as purple dashed lines.

To understand the molecular details of how our Iteration 3 designed antibody, Ab#10-M30W-S94M, recognizes BA.4/5 RBD, we determined a cryo-EM structure of the BA.4-S/Ab#10-M30W-S94M IgG complex. The resolution of the global density map was 3.1 Å (Fig. 6b, Extended Data Fig. 6 and Extended Data Table 1). The RBD regions that were bound to Ab#10-M30W-S94M Fabs were not well-resolved. We therefore employed a local density map refinement strategy and we obtained a well-resolved local density map corresponding to BA.4-S RBD and the variable domains V_H_ and V_L_ of Ab#10-M30W-S94M at a resolution of 3.6 Å (Fig. 6c, Extended Data Fig. 6 and Extended Data Table 1). As expected, two RBDs are in “up” positions and one RBD is in “down” position (Fig. 6a).

In our subsequent analysis of the BA.4-S/Ab#10-M30W-S94M epitope/paratope interface, we identified the key interactions to improved binding and neutralization potency. First, D100_D_N on the antibody heavy chain forms h-bonds with side chains of BA.4-S RBD N487 and antibody light chain G50Y. Light chain G50Y and S31M further interact with BA.4-S RBD T478K side chain C_β_-C_ε_ by hydrophobic interactions. This hydrophilic-hydrophobic interaction core further locks the side chain position of BA.4-S RBD T478K of which the ε-amine interacts with the backbone carbonyls of light chain I48 and Y49 via h-bonds (Fig. 6d). Another new hydrophobic interaction is formed between light chain S93G, S94M and RBD E484 (Fig. 6e). Lastly, a critical hydrophobic core is formed between antibody heavy chain M30W, G53A and BA.4-S RBD F456, Y489. Additionally, the heavy chain N56E side chain and S54 backbone carbonyl form h-bonds with the BA.4-S RBD R493Q side chain (Fig. 6f).

In summary, by applying Rosetta single-position NNK scanning, we successfully designed a 2196 antibody variant—Ab#10-M30W-S94M— that restores neutralization to BA.4/5 variants, BA.1/2 variants and preserves neutralization to the WA variant. Our cryo-EM structure shows that multiple new stabilizing interactions were introduced providing the molecular basis for improved Ab#10–30W-94M binding and restored neutralization potency.

## Discussion

Since COVID-19 emerged, many therapeutic antibodies have been characterized and deployed. However, the emergence of SARS-CoV-2 Omicron subvariants, particularly BA.1, BA.2, and BA.4, BA.5 challenged the effectiveness of previously potent monoclonal antibodies due to numerous RBD mutations that disrupted critical antibody-RBD interactions^15^. Here, we present a structure-based, iterative and fast design strategy to systematically modify the COV-2019 antibody to restore its neutralization potency against these Omicron variants while retaining neutralization potency for the parental WA variant. The design workflow leveraged structural modeling, Rosetta-based computational design, experimental and cryo-EM validation to iteratively improve antibody paratope compatibility with mutated RBD epitopes. Our design protocol requires minimal computational resources and is a precise platform identifying optimal and efficacious antibody designs.

Our Iteration 1 design was focused on compensating for key lost interactions between 2196WT and the BA.1/2 RBD, particularly those disrupted by T478K and E484A mutations. Substitutions G50L and S93F/Y on the light chain of 2196WT showed measurable improvement in binding to BA.1/2 RBD and neutralization to BA.2 pseudoviruses. Structural characterization confirmed the formation of novel interactions at the epitope/paratope interface, suggesting that these changes partially alleviated the loss of contacts caused by Omicron BA.1/2 RBD mutations. Notably, these designs resulted in improved IC50 neutralization values against BA.2 pseudovirus, yet the restoration was only partial and did not fully recapitulate ancestral-level potency, suggesting that our compensatory mutations could mitigate but not entirely reverse immune evasion by highly mutated RBDs.

Our Iteration 2 efforts, based on our refined atomic-resolution cryo-EM structures from Iteration 1, expanded mutational targeting to 11 positions and aimed to introduce additional stabilizing interactions. The top candidate, Ab#10, achieved lower IC50 neutralization values than its parental constructs and acquired modest binding to BA.4/5 RBD, indicating partial success in expanding variant coverage. Mutational reversals combining Iteration 1 and 2 designs impaired function, highlighting that the functional impact of a mutation can depend heavily on the specific antibody context in which it occurs. These findings underscore the inherent complexity in optimizing multiple interdependent antibody residues.

Despite the promising in vitro neutralization potency of Ab#10 against BA.2, its limited activity against BA.4 highlighted the adaptive limits of prior designs. Iteration 3 design therefore focused specifically on BA.4/5 variants, incorporating additional mutations targeting the disrupted hydrophobic core and interface changes introduced by BA.4/5-specific substitutions (e.g. F486V). A comprehensive mutation scanning approach guided the selection of interface positions for optimization, leading to the identification of Ab#10-M30W-S94M. This antibody design exhibited substantial improvement in neutralization potency against both BA.2 and BA.4 pseudoviruses while maintaining neutralization potency for WA pseudovirus with IC50 values against all these variants being similar to IC50 of 2196WT against the ancestral WA strain. Consistent with clinical validation of DNA-encoded monoclonal antibody delivery, where 39/39 participants demonstrated sustained in vivo expression without anti-drug antibody (ADA) responses, the neutralization profile of Ab#10-M30W-S94M against BA.4 supports its translational potential for DNA-based delivery^21^. The structural basis for the restored BA.4 binding and neutralization by Ab#10-M30W-S94M was examined via cryo-EM, revealing that the new paratope mutations effectively accommodated epitope alterations and created new key interactions.

Importantly, the entire design process could be carried out using standard CPU-based computational resources, demonstrating that effective antibody optimization does not require large-scale infrastructure or high-performance computing. This accessibility broadens the potential for rapid and responsive therapeutic development, particularly in settings with limited resources. Although further in vivo validation is needed to assess clinical efficacy, our findings highlight a streamlined and adaptable antibody engineering strategy that could complement current efforts to manage ongoing viral evolution.

Our results emphasize the non-linear nature of antibody engineering. Mutations that improve binding in one design background may be detrimental in another, even if they target the same epitope region. This underscores the role of structural context and intramolecular interactions in shaping the effect of paratope mutations. Furthermore, although we employed ΔΔG energy-based ranking as selection criteria, these metrics do not directly predict neutralization potency, and experimental validation remains indispensable.

Future directions include expanding the design scope to include additional antibody families and variant targets, validating efficacy in authentic viral infection models, and integrating predictive modeling of antibody properties such as manufacturability, solubility, and immunogenicity. Ultimately, the lessons learned from this study contribute to a growing body of work suggesting that computationally guided antibody design is a powerful approach to sustaining therapeutic relevance amid rapid viral antigenic shifts.

## Materials and methods

### Rosetta multi-position design for Iterations 1 and 2

To obtain the lowest energy state for the design procedure, the original atomic models were first processed through Rosetta FastRelax^22^. After 100 repacking and energy minimization runs, the resulting lowest energy score atomic model was used as input structure for design. Prior to design, ΔG_predesign_ of binding was calculated by way of InterfaceAnalyzer by subtracting “post_dG_separated” from “pre_dG_separated”. Identified positions were designated as all amino acids or restrained to polar amino acids or nonpolar amino acids as described in the Results section. Neighboring residues within 6Å of the selected design positions were repacked and energy minimized. ΔG_postdesign_ of binding was then calculated by way of InterfaceAnalyzer by subtracting “post_dG_separated” from “pre_dG_separated”. ΔΔG for each design was then calculated as ΔG_predesign_ subtracted from ΔG_postdesign_. For Iteration 1 design, our BA.2-RBD/2196WT model was relaxed using FastRelax and 120 decoys were designed and for Iteration 2 design, our BA.2-RBD/2196-G50L-S93F structure was relaxed using FastRelax and 3000 decoys were designed.

### Single-Point Mutational ΔG Binding Energy Analysis for Iteration 3 design

To identify potential mutations that could enhance the binding affinity between Ab#10 and the BA.4 SARS-CoV-2 receptor-binding domain (RBD), we performed a single-point mutational scan across the paratope/epitope interface to estimate the ΔG contribution to binding energy of each point mutation, defined as the binding energy of the wild-type complex minus that of the point-mutated complex. We first utilized the previously solved structure of the BA.2-RBD/2196-G50L-S93F complex and generated a BA.4-RBD/Ab#10 atomic model by performing a fixed-backbone design, introducing all necessary mutations, and repacking side chains. The remodeled complex was subsequently relaxed using a RosettaRelax constrained relax protocol. Single-point mutational scanning was then conducted at selected positions to evaluate changes in binding energy upon mutation. Each mutation was introduced using a fixed-backbone protocol, with residues within a 6 Å radius subject to side-chain repacking. The resulting ΔG of binding were compiled in a CSV file, and a heatmap was generated for downstream evaluation.

### Genes and expression plasmids

Designed antibodies heavy chain and light chain gene fragments were generated by synthesized (pTwist) or single mutation mutagenesis. Synthesized gene fragments were cloned in the pVax expression vector (Thermofisher). WA-S protein (derived from isolate USA/WA1/2020) with 6 proline mutations (6P)^23^, BA.1-S (Omicron sublineage BA.1.1.529) with 5P, also BA.4-S (Omicron sublineage BA.4) with 5P and a disulfate modification (V720C and T898C) were subcloned in the pVax expression vector. BA.1/2 RBD and BA.4/5 RBD expression plasmids were purchased from Addgene (#184830 and #213478). All DNA for transfection was prepared using Midi Xtra Midi Plus EF (Takara).

### Protein production and purification

Epxi293 and Freestyle293F cells (Thermofisher) were cultured in Expi293 culture medium and Freestyle293 culture medium, respectively. Cells were maintained in suspension culture at 37°C, 5% CO2, humified incubator shaking at 215 rpm. Purified IgG DNA or spike DNA were transfected into Expi293 cells. BA.1/2 RBD and BA.4/5 RBD DNA were transfected into Freestyle 293 cells. All transfections were conducted using FectoPro (Polyplus) according to the manufacture protocol. Supernatant of transfected IgGs were incubated with ProteinA Fast Flow resin (Cytiva) overnight at 4°C. ProteinA resin was washed with PBS buffer (pH 7.2) followed by elution with 0.1M Glycine-HCl (pH 3.5). Eluted fractions were neutralized immediately with 1M Tris-HCl. Purified IgGs were buffer exchanged to PBS buffer. Supernatant of transfected spike proteins were incubated with Lectin resin (VectorLabs) overnight at 4°C. Resin was washed with PBS buffer (pH 7.2) followed by elution with 1M Methyl α-D-mannopyranoside in PBS buffer. Spike proteins were further purified via Superose 6 Increase 10/300 column (Cytiva) in PBS buffer. RBD proteins were first affinity purified on a HiTrap IMAC column (Cytiva) according to the manufacture protocol. Specifically, the supernatant was applied to the IMAC column followed by washing the column with 25mM Imidazole, PBS buffer. RBD proteins were eluted by gradient elution buffer from 25mM Imidazole to 1M Imidazole PBS buffer. Affinity purified RBD proteins were further purified through Superdex 200 Increase 10/300 column (Cytiva) in PBS buffer.

### Enzyme-linked Immunosorbent Assay (ELISA)

SARS-Cov-2 RBD proteins (BA.1/2 and BA.4/5) were pre-coated overnight at 4°C in PBS at 2ug/mL in flat-bottom 96-well half-area plates (Corning). After washing off the extra RBD protein using wash buffer (PBS, 0.1% Tween-20) three times, plates were blocked in blocking buffer (PBS, 0.1% Tween20, 5% skin milk) for 2 hours at room temperature. IgG proteins, positive control (TH132)^24^, negative control (HIV bNAb, 8ANC195)^25^ and background control (blocking buffer) were serially diluted at 100ug/mL using a 5-old dilution series in blocking buffer followed by incubation with the pre-coated plates for 1 hour and 30 minutes. Three-times washed plates were incubated with 10,000-fold diluted secondary Goat Anti-human IgG-Fc HRP conjugated antibody (Bethyl Laboratories) for 45 minutes. After washing plates three times with washing buffer, the reactions were developed by incubating the plates with the TMB ELISA substrate solution (Thermofisher) for 10 minutes followed by adding 1M H_2_SO_4_ to stop the reaction. The absorbance was recorded using a Biotek Synergy 2 plate reader at 450nm and 570nm. Absorbance at 570nm served as background absorbance and was subtracted from each data point. Using GraphPad Prism 10, each sample curve was fitted using a non-linear regression model.

### Pseudovirus Neutralization assay against various strains

SARS-CoV-2 spike protein pseudotyped viruses are capable of only one round of infection after entry into cell lines with appropriate receptors required for infection. For production of SARS-CoV-2 pseudoviruses, HEK293T were maintained in DMEM (Corning) supplemented with 10% fetal bovine serum (FBS) (Peak Serum, PS-FB2) at 37°C and 5% CO_2_. Gene jammer (Agilent) was used to transfect HEK293T cells with 1:1 ratio of pNL4–3.Luc.R-E-plasmid (NIH AIDS reagent Program) along with plasmids of various env strains (Genscript) expressing the parental spike protein (derived from isolate USA-WA1/2020), B.1.1.529/BA.2 (Omicron sublineage BA.2) and B.1.1.529/BA.4 (Omicron sublineage BA.4). Forty-eight hours post-transfection, culture supernatants were collected, enriched with FBS to 12% final volume. huCHOAce2 cells (Creative Biolabs; VCeL-Wyb019) were grown in tissue culture and then inoculated with various dilutions of the virus of interest in order to determine their titer and dilution of stock required to yield a minimum of 20 times RLU obtained in cells, where no virus was added.

SARS-CoV-2 pseudovirus neutralization assays were established by using huCHOAce2 cells as reported earlier^21^. Briefly, 10,000 cells/well were plated in 96-well plates in sterile 96-well flat-bottomed tissue culture-treated plates (Falcon) in D10 media (DMEM supplemented with 10% FBS Cytiva-cat#SH30406.02HI and 1X Penicillin-Streptomycin; Corning) and incubated overnight in 37°C and 5% CO_2_. The following day, samples were serially diluted and incubated with psedoviruses for 90 min at room temperature before transfer to huCHOAce2 cells. MAbs 2130 and 2196 obtained from AstraZeneca served as controls. Plates were further incubated at 37°C andt 5% CO_2_ for 72 h and then lysed using the britelite plus luminescence reporter gene assay system (cat. no. 6066769, Revvity). Luminescence was measured using the Biotek Synergy 2 plate reader. Using GraphPad Prism v.10, each sample curve was fitted using a non-linear regression (log-inhibitor vs. normalized response – variable slope) with a constraint of Hill slope < 0. Minimal infective dose (ID50) was then calculated, defined as the reciprocal of the dilution required to achieve 50% neutralization in RLU. IC50 values were derived by dividing the corresponding IgG titer (ng/mL) by the ID50.

### Cryo-EM sample preparation and data collection

BA.1-S/2130 IgG, BA.1-S/2196 IgG, BA.1-S/2196-S93Y, BA.1-S/2196-G50L-S93F, BA.1-S/2196-S93Y/2130WT and BA.1-S/2196-G50L-S93F/2130-S30_B_G and BA.4-S/Ab#10-M30W-S94M complexes were made by incubating spike proteins with corresponding IgG proteins (2196 variants) or Fab proteins (2130 variants) at 1:9 or 1:12 molar ratio on ice for 2 hours followed by purification via Superose 6 Increase 10/300 column. Corresponding complex fractions were concentrated at 1mg/mL. BA.1-S/2130 IgG, BA.1-S/2196-S93Y, BA.1-S/2196-G50L-S93F, BA.1-S/2196-S93Y/2130WT, BA.1-S/2196-G50L-S93F/2130-S30_B_G and BA.4-S/Ab#10-M30W-S94M complexes were applied to freshly glow-discharged UltrAuFoil 1.2/1.3 or Au-Flat 1.2/1.3 grids (Quantifoil, Electron Microscopy Sciences) in a Vitrobot Mark IV (Thermofisher) at 4°C, 100% humidity. BA.1-S/2196 IgG complex was prepared similarly but applied on in-house made graphene oxide coted UltrAuFoil 1.2/1.3 grid. After blotting off excess sample, grids were plunge-frozen in liquid nitrogen cooled liquid ethane.

Data collection for all samples were performed on a Titan Krios G4 (Thermo Fisher Scientific) equipped with a Gatan K3 detector at 105,000 magnification. All data sets were collected at ~ 45–60 e-/Å2 across 45–50 frames. The defocus range was − 0.5μm-2.5μm. Data processing for all data sets were done similarly. Raw movies were imported to Relion^26^ followed by Motion Correction and CTF estimation^27^. Particles were picked using blob picker and then extracted. 2D classification was performed followed by Ab-initio 3D object reconstruction in CryoSPARC^28^. Heterogenous refinement (CryoSPARC) was then performed and converging data classes were refined using non-uniform refinement in CryoSPARC. CTF-refinement and Bayesian polishing (Relion) were then performed and the resulting data classes were subject to a final round of non-uniform refinement in CryoSPARC. Particle subtraction was performed using a mask excluding the RBD-Fab part, then local refinement was conducted resulting in the final local density maps.

### Model building

Initial model used in this study was WA-RBD/2196/2130 (PDB 8D8Q). Reference models were docked into the density maps in UCSF ChimeraX^29^ and manually rebuilt and real-space refined in Coot^30^. Models were then refined using Rosetta FastRelax in real space allowing Cartesian degrees of freedom and rebuilt and real-space refined in Coot iteratively. Final protein model geometry was validated by MolProbity^31^, glycan geometry by Privateer^32^ and model fit-to-map was validated by EMRinger^33^.

## Supplementary Material

This is a list of supplementary files associated with this preprint. Click to download.

- ExtendedDataTable1.xlsx
- ExtendedDatacompressed.pdf
